# The Impact of Women’s Opportunity Costs on Household Fertility Decisions: Evidence from China

**DOI:** 10.3390/bs16060930

**Published:** 2026-06-05

**Authors:** Jingfeng Xu, Laile Tang, Qijun Huang, Xiaojia Wang

**Affiliations:** School of Insurance, Central University of Finance and Economics, Beijing 100081, China

**Keywords:** population sustainability, women’s opportunity costs, life-cycle model

## Abstract

As a core component of childbearing costs, women’s opportunity costs provide a crucial perspective for explaining the current decline in fertility rates. Recognizing the reciprocal causality between women’s opportunity costs and fertility decisions, this study examines their statistical correlation using micro-level data from the China Family Panel Studies (CFPS). Building on these empirical insights, we develop a household fertility decision-making model that incorporates women’s opportunity costs, calibrating the parameters through structural estimation to quantitatively explore its impact on fertility choices. The quantitative empirical findings reveal a significantly negative correlation between women’s opportunity costs and the actual number of children in a household. The theoretical analysis demonstrates that an intensifying motherhood penalty and prolonged career interruptions due to childbirth both lead to a reduction in the equilibrium number of children. Furthermore, higher educational attainment and increasing child-rearing costs exert a pronounced inhibitory effect on fertility intentions. Policy simulations further indicate that, compared to short-term or one-off incentives, continuous fertility subsidies and the implementation of free childcare policies are more effective in offsetting opportunity costs and boosting household fertility intentions.

## 1. Introduction

The persistent decline in China’s birth rate has drawn widespread attention in recent years, resulting in increasingly severe socio-economic challenges such as population aging that hinder economic growth (Zhong, 2011; Liang et al., 2018). To address this trend, China has gradually relaxed its fertility policies, yet their effectiveness remains limited. The nationwide implementation of the two-child policy in 2016 temporarily increased fertility rates, but failed to substantially reverse the long-term downward trajectory. According to the China Statistical Yearbook 2024, the national birth rate declined from 11.99 per thousand in 2015 to 6.39 per thousand in 2023, with transient rebounds to 13.57‰ (2016) and 12.64‰ (2017) post-policy adjustment.

Childbearing costs are a critical determinant shaping household fertility decisions, encompassing both quantifiable objective financial expenditures and subjective psychological burdens that are challenging to measure. Within the conventional economic framework, fertility decisions are predominantly viewed as a rational trade-off involving economic costs. High fertility costs constitute a primary constraint on household childbearing decisions. Leibenstein (1957) developed a microeconomic model of fertility decision-making, categorizing fertility costs into direct expenditures (explicit costs such as childcare and education) and opportunity losses (implicit costs like career interruptions). His findings indicate that, as household income grows, the rate of increase in childrearing costs surpasses the marginal utility derived from additional children, thereby significantly suppressing fertility intentions. Becker and Tomes (1976) integrated fertility decisions into consumer choice theory, further decomposing costs into fixed expenditures (basic childrearing expenses) and variable inputs (discretionary investments in education and healthcare). His research demonstrates that when household income exceeds a critical threshold, rational decision-makers reduce fertility quantity to intensify human capital investment in fewer offspring, establishing this as the core mechanism explaining low fertility in modern societies.

A growing body of literature indicates that individuals consistently exhibit bounded rationality in decision-making (Simon, 1955) and respond to subjective perceived constraints rather than objective ones (Tversky & Kahneman, 1981). To this end, behavioral economics and social psychology have further expanded the research boundaries of fertility decision-making, suggesting that fertility choices are no longer driven solely by economic factors. The theory of work–family role conflict posits that when women confront the dual role conflict between family child-rearing and career advancement, the resulting psychological costs, such as anxiety and tension, simultaneously constitute an important implicit barrier impeding fertility decisions (Greenhaus & Beutell, 1985; Netemeyer et al., 1996; Brewster & Rindfuss, 2000; Begall & Mills, 2011). From the perspective of identity economics theory, because society maintains divergent career expectations for men and women (Akerlof & Kranton, 2000; Bertrand et al., 2015; Elitzur & Solodoha, 2021; Solodoha et al., 2026), childbearing also carries hidden costs, such as career trajectory interruptions and the weakening of professional self-identity. Simultaneously, the social signals received by households and their idiosyncratic personal traits may also shape their decision-making behaviors (Solodoha, 2024; Connelly et al., 2011; Teti & Gelfand 1991; Chernyak-Hai & Solodoha, 2026). Furthermore, the theory of planned behavior proposed by Ajzen (1991) emphasizes the roles of attitudes, subjective norms, and perceived behavioral control in shaping individual choices, providing an analytical paradigm for a substantial body of subsequent research. Taking Bulgaria as a case study, Billari et al. (2009) conducted a sample survey based on TPB; their results demonstrated that the three components of the theory can largely predict fertility intentions, and that attitudes are more relevant than norms for higher parities. Applying TPB to the study of fertility decisions, Ajzen and Klobas (2013) focused on evaluating how the three antecedent factors—attitudes, subjective norms, and perceived behavioral control—affect fertility decisions, while thoroughly exploring the background influences of institutional policies, social values, and individual characteristics.

Among the aforementioned multidimensional childbearing costs, women’s opportunity costs constitute a major component of household fertility constraints. These costs also serve as the tangible, labor-market manifestation of the behavioral role conflicts and identity-based tensions discussed above. Ignoring these costs in fertility cost analyses would lead to biased conclusions. Becker (1960) established the “New Home Economics” framework, pioneering the integration of female time value into fertility decision-making models. He posited that rising female wage rates elevate the opportunity costs of childbearing, thereby reducing fertility rates. Willis (1973) incorporated household production functions and dynamic human capital investments into fertility models, proposing that childbearing reflects a “quantity–quality trade-off” under parental time constraints. His research demonstrated that increased maternal wages suppress fertility through heightened opportunity costs, whereas the interaction between husband’s income and female education generates a U-shaped fertility curve. Kleven et al. (2019) revealed that the motherhood penalty is the core driver of the gender earnings gap. They demonstrated that women’s behavioral choices after childbirth (such as a tendency to select more secure, slower-advancing “family-friendly” positions) are heavily influenced by the intra-household division of labor demonstrated by their own mothers. This subconscious preference dependency constitutes a long-term career progression opportunity cost for women. Bertrand et al. (2015), grounding their study in identity economics theory, found that traditional gender identity norms exert strong constraints, with societal expectations of the maternal role being further intensified following childbirth. To align with their identity perceptions, highly skilled women often proactively reduce their labor supply and forgo career advancement opportunities, thereby incurring substantial implicit career opportunity costs. Furthermore, utilizing micro-level data from Denmark, Barigozzi et al. (2024) employed behavioral economic modeling by incorporating social norms directly into the utility function. This framework elucidates the underlying mechanisms connecting social norms, gender-specific career choices, and child-rearing decisions, as well as the potential opportunity costs that childbearing behavior imposes on women’s career paths.

With the continuous deepening of research on women’s opportunity cost, a large body of literature has begun to conduct multidimensional empirical quantifications centering on the “motherhood penalty,” which serves as the objective manifestation of women’s opportunity costs. Polachek (1985) posited that women experience career interruptions post-childbirth, leading to reduced personal productivity. Neumark and Korenman (1992), Waldfogel (1997), and Budig and England (2001) conducted quantitative studies using data from the United States and the United Kingdom, revealing a persistent 7–10% wage rate reduction per additional child, indicating widespread fertility-related economic penalties. Adda et al. (2017) found that women with children earned 5% more than childless women at age 20, but this advantage reversed by age 40, with childless women earning 10% more. Their analysis identified career interruptions and associated long-term income losses due to childbearing as the core mechanism driving expanded income disparities among women of reproductive age. Jia and Dong (2013) examined how China’s economic transition influenced wage gaps between urban mothers and childless women using panel data from 1990 to 2005. The results revealed substantially lower incomes for mothers compared to childless women, with the motherhood wage penalty intensifying over time.

Concurrently, a robust negative association between women’s opportunity cost and diminished fertility intentions has been widely documented across a substantial body of literature. Salamaliki et al. (2013) analyzed U.S. data from 1948 to 2007, identifying a negative correlation between female labor force participation and fertility rates, thus validating Becker’s (1993) New Home Economics framework that measures women’s opportunity costs through wage income, in which higher wages correspond to lower fertility. Huinink and Kohli (2014) integrated implicit career development costs into life-cycle models, demonstrating that rising opportunity costs (income loss, limited promotions) significantly delay or reduce fertility, particularly in societies lacking supportive policies like childcare infrastructure. Choi (2017) incorporated abortion decisions into fertility models, showing that abortion likelihood depends on the trade-off between opportunity costs and perceived child utility. Calibrating population characteristics, the study revealed that lower contraceptive efficacy drives higher abortion rates among high school populations, whereas higher-asset households exhibit lower abortion demand due to greater capacity to absorb fertility costs. Furthermore, according to the loss aversion logic of prospect theory, individuals weigh perceived losses significantly more heavily than equivalent gains (Kahneman & Tversky, 2013). This asymmetry causes the anticipated losses associated with childbearing, such as setbacks in career progression and personal growth, to be disproportionately amplified, meaning their inhibitory effects on fertility decisions may very well exceed that of measurable economic income effects (Liefbroer, 2005).

While the existing literature has predominantly utilized empirical analyses to investigate the negative impacts of childbearing on women’s wage rates and employment status, as well as the robust statistical correlation between women’s opportunity cost and diminished fertility intentions, no studies to date have quantitatively modeled these dynamics through calibrated life-cycle frameworks. This study bridges this gap through a dual-method approach. Serving as the empirical foundation for our theoretical analysis, the empirical results document a robust negative association between women’s opportunity cost and the number of children in a household, suggesting that such costs play a significant role in shaping household fertility decisions. Next, drawing upon the lifecycle models of Rees and Scholz (2010), Bick (2016), and Sommer (2016), we develop a joint household consumption–saving–fertility decision-making model. In this framework, women’s opportunity costs of childbearing are characterized by the potential income risk they face following childbirth. Following the parameter calibration methodologies of DellaVigna (2018), Gill and Prowse (2012), and Laibson et al. (2026), we employ structural estimation to calibrate individual subjective preference parameters, thereby enhancing the behavioral realism of the model.^1^ Finally, by constructing various policy simulation scenarios, we quantitatively investigate household fertility choices under the constraints of women’s opportunity costs.

Specifically, in the empirical analysis, this study employs data from the 2022 data of China Family Panel Studies (CFPS), using the number of children per household as the dependent variable and career interruption due to childbearing (as a measure of opportunity costs) as the explanatory variable to investigate the impact of women’s opportunity costs on household fertility decisions. The empirical results demonstrate a significant negative correlation between household fertility decisions and women’s opportunity costs: the higher the opportunity costs of childbearing for women, the fewer children families ultimately choose to have, with an increased tendency toward childlessness. In the theoretical analysis, this study integrates women’s opportunity costs and rigid childrearing expenditures into a life-cycle model, constructing a household consumption–saving–fertility decision-making framework that characterizes opportunity costs as potential lifelong wage reduction caused by childbearing. When calibrating parameters through structural approaches, we apply unbiased estimation to precisely measure objective parameters, such as income levels and child-rearing expenditures. Conversely, for subjective parameters, including the coefficient of relative risk aversion and the degree of preference for children, we employ the simulated method of moments (SMM) based on individual behavioral traits. This strategy allows the model to effectively capture the behavioral drivers underlying household fertility decisions. After parameter calibration, the theoretical model validates the hypotheses and conclusions derived from the empirical analysis. Scenario simulations further quantify the effects of other influencing factors and policy interventions on household fertility decisions. Through these empirical and theoretical analyses, this study provides a comprehensive quantitative examination of the impacts of women’s opportunity costs on household fertility decisions, offering both theoretical and empirical foundations for optimizing fertility support policies.

The marginal contribution of this study lies in extending the life-cycle models of Rees and Scholz (2010), Bick (2016), and Sommer (2016) by innovatively incorporating women’s opportunity costs into the life-cycle decision-making framework. Concurrently, drawing upon the parameter calibration methodologies of DellaVigna (2018), Gill and Prowse (2012), and Laibson et al. (2026), this study employs structural estimation to calibrate the subjective preference parameters embedded within fertility decision-making. Utilizing the calibrated model, we quantitatively analyze how women’s opportunity costs, income, and other expenses influence household fertility decisions.

The remainder of this paper is structured as follows: Section 2 presents quantitative facts, Section 3 constructs the theoretical model, Section 4 discusses parameter calibration, Section 5 provides the results and analysis, and Section 6 concludes the study.

## 2. Quantitative Facts

This study aims to explore the impact of opportunity costs on household fertility decisions. This section first conducts an empirical analysis based on micro-level survey data to examine the relationship between women’s opportunity costs and the number of children in the household, which subsequently serves as the foundation for the theoretical modeling in the following sections.

### 2.1. Data Sources

This study uses households as empirical analysis units, with microdata sourced from the CFPS 2022 and regional macroeconomic data (GDP) obtained from the China Statistical Yearbook.

Following research objectives and referencing prior literature (Ajzen & Klobas, 2013), we operationalize women’s opportunity costs using career interruptions caused by childbearing as the proxy variable.^2^ By integrating considerations of optimal childbearing age and working-age participation, the study focuses on married women aged 20–50 years with childbirth experience as the target population to analyze how women’s opportunity costs affect household fertility decisions. To account for multidimensional influences on fertility intentions, we additionally matched and screened variables related to maternal (i.e., married and parous women), paternal, household, and regional characteristics. Based on the CFPS 2022 dataset, observations with missing values in the variables used in the analysis were excluded, resulting in a final sample of 1243 households.

### 2.2. Model Specification and Variable Selection

To investigate the impact of women’s opportunity costs on fertility decisions, the following baseline regression model was constructed:^3^

(1) $$child_{{num}_{i}}=\beta_{0}+\beta_{1}{opportunity}_{i}+\beta_{2}X_{i}+\lambda_{p(i)}+\varepsilon_{i}$$

where $child_{{num}_{i}}$ represents the actual number of children in the i-th family; ${opportunity}_{i}$ denotes the opportunity cost for women in the household,^4^ i.e., the loss borne by the mother due to fertility; and $X_{i}$ represents control variables, including female characteristics, spouse characteristics, household characteristics, and regional characteristics. Here, $\lambda_{p(i)}$ denotes province fixed effects and $\varepsilon_{i}$ represents the error term.

Specifically, regarding women’s individual characteristics, this study includes women’s age, educational attainment, and health status to control for differences in life-cycle stage, human capital accumulation, and labor supply capacity (Becker, 1981; Klesment & Van Bavel, 2017; Case et al., 2005). With respect to spousal characteristics, spouse’s educational attainment and health status are incorporated to capture household resource endowment and the structure of household labor supply (Mare, 1991). At the household level, household income, government subsidies, and household debt are included to account for household financial constraints and economic pressure (Becker & Lewis, 1973; Lusardi & Tufano, 2015; Aassve et al., 2021). Regarding regional characteristics, in addition to urban–rural status and regional GDP per capita, province fixed effects are further introduced to control for regional heterogeneity in economic development, labor market conditions, fertility culture, and public service provision across provinces (Lesthaeghe, 2010; Doepke et al., 2023).

Detailed definitions of the variables and the descriptive statistics are reported in Table 1. In addition, the multicollinearity test results indicate that the model does not suffer from severe multicollinearity problems.

Table 1 shows that, for families meeting the age restrictions, the average number of children per woman in the sample is 1.619. Specifically, 48.35% of families have one child, 43.28% have two children, and 8.37% have three or more children. According to a 2021 survey by the National Health Commission, the fertility intentions of women of childbearing age continue to decline, with an average intended number of children being 1.64. This indicates that the sample is reasonable and representative.

The mean value of women’s opportunity costs in the sample is 0.051, suggesting that approximately 5% of women stopped working due to fertility, childcare, or other family-related reasons. The highest educational level among women is a master’s degree, whereas the lowest is no formal education, with an average of 11.274 years of education. For spouses (husbands), the highest educational level is a doctoral degree, with an average of 11.505 years of education, slightly higher than that of women.

### 2.3. Empirical Results

Table 2 presents the baseline regression results.

Table 2 shows that the regression coefficient for female fertility opportunity costs is −0.1547, which is statistically significant at the 5% level. The results indicate a statistically significant negative relationship between women’s opportunity costs and the number of children in the household. For women, especially those with high incomes or high professional achievements, childbearing may lead to career interruptions, reduced promotion opportunities, or even workplace discrimination. These factors increase both the economic and psychological costs of childbearing, thereby suppressing the actual number of children in a household. It should be noted, however, that this result primarily identifies a conditional correlation between women’s opportunity costs and the number of children in the household, rather than a strict causal effect on fertility decisions.

Additionally, Table 2 reveals three key findings. First, households in which women have longer years of education tend to have fewer actual children. On one hand, education improves labor market opportunities and increases time costs; as women’s years of schooling increase, their opportunity costs of childbearing tend to increase. On the other hand, longer educational attainment delays graduation and entry into the labor market, which subsequently leads to later marriage and delayed childbearing, resulting in fewer actual children. Second, households with higher per capita income tend to have fewer actual children. Families with higher income levels tend to reduce fertility to allocate more resources toward children’s education and living quality. At the same time, high-income groups face greater childcare opportunity costs and stronger career pressures, which further reduces their realized fertility. Third, urban households tend to have fewer actual children. Urban families face higher child-rearing costs, and women in urban areas incur higher opportunity costs associated with childbirth. As a result, these households are more likely to reduce their number of actual children.

### 2.4. Robustness Tests

To ensure the robustness of the baseline results, this study conducts a series of robustness checks on the baseline model.

First, the observation range of the father’s age in the household is narrowed from the original unrestricted range to the fertility group aged 22–50 years. The regression results are reported in column (1) of Table 3. On the one hand, men under 50 years of age are the main group for fertility, and the phenomenon of “having children at an older age” is not common in practice; on the other hand, narrowing the observation range to the fertility group under 50 years of age helps to reduce age-related heterogeneity, as fertility decisions in younger groups are more likely to be influenced by similar social, economic, and cultural factors.

Second, this study replaces the regression model and sequentially uses the ordered Probit model, the ordered Logit model, and the Poisson regression model for robustness testing. The results of the robustness tests are shown in columns (2)–(4) of Table 3.

Third, based on the baseline regression model, we further include a control variable indicating whether elderly family members aged 60 years and above reside in the household. This is intended to better account for labor market withdrawal induced by caregiving responsibilities for the elderly, thereby mitigating potential confounding effects from non-childbearing-related household duties. The corresponding results are reported in column (5) of Table 3.

Fourth, we replace the key explanatory variable by constructing alternative continuous measures of women’s opportunity costs; namely, the duration of career interruptions and the implied wage penalty. These alternative proxies are used to capture women’s opportunity costs in a more refined manner, and the regression results are reported in columns (6) and (7) of Table 3.

According to the robustness results reported in Table 3, there remains a negative relationship between women’s opportunity costs and the number of actual children in the household. This is consistent with the baseline results, suggesting that the findings are robust.

In addition, this study further conducts heterogeneity analyses across subsamples, including urban–rural heterogeneity and heterogeneity by women’s educational attainment. The corresponding regression results are reported in Table 4.

Based on columns (1) and (2) of Table 4, the urban–rural heterogeneity results show that in the urban subsample, the coefficient on women’s opportunity costs is −0.1843 and is statistically significant at the 5% level. This indicates a pronounced negative association between women’s labor market exit due to childbirth or family responsibilities and the number of actual children in urban households. In contrast, although the coefficient on opportunity costs is also negative in the rural subsample, it is not statistically significant. This suggests that, compared with rural areas, the negative relationship between women’s opportunity costs and fertility behavior is more pronounced in urban households. This pattern may be attributed to higher female labor force participation, greater opportunity costs of career advancement, and more intense competition for childcare resources in urban areas.

Based on columns (3) and (4), the heterogeneity analysis by educational attainment shows that in the subsample with higher education levels, the coefficient on women’s opportunity costs is −0.2157 and is statistically significant at the 5% level. In contrast, although the coefficient remains negative in the lower education subsample, it is not statistically significant. This finding suggests that the negative association between women’s opportunity costs and fertility is relatively stronger among more educated households. A possible explanation is that highly educated women typically possess greater human capital accumulation and stronger career prospects; therefore, the income loss, career interruption, and time reallocation costs associated with childbearing are relatively higher, leading them to further reduce fertility.

Overall, across both urban–rural and educational attainment subsamples, the estimated coefficients of women’s opportunity costs are consistently negative, indicating a robust negative relationship between women’s opportunity costs and the number of children in the household, consistent with the baseline regression results.

## 3. Theoretical Model

The aforementioned empirical analysis and robustness checks reveal a robust negative association between women’s opportunity cost and the actual number of children in a household, thereby establishing a solid empirical foundation for the theoretical analysis of this study. Building on these findings, this study incorporates women’s opportunity costs into a fertility decision-making model to quantitatively analyze their effects. The model operates under four assumptions. First, childbearing exposes women to uncertain opportunity costs, which may lead to a reduction in future earnings. Second, households face rigid childrearing costs spanning childbirth, childcare, education, and housing. Third, spousal incomes are subject to periodic uncertainty and potential income shocks. Fourth, spouses share identical preferences and jointly determine household consumption, savings, and fertility decisions to maximize the household’s lifetime expected utility.

This study assumes that, at the initial time, both spouses are of the same age (x years old), and their maximum age is $x_{T}$ years old, with no risk of premature death during this period. Considering the age window for fertility, the wife’s maximum fertility age is $x_{F}$ years old, beyond which she can no longer have children. The husband retires at age $x_{R}^{M}$, and the wife retires at age $x_{R}^{F}$.

### 3.1. Income Process and Women’s Opportunity Costs

#### 3.1.1. Income Process

This study assumes that during the working period, household income consists of two components: the labor income of the male (husband) and the labor income of the female (wife).

Following Liao and Liu (2025) and Cho et al. (2021), this study assumes that individual labor income is influenced by deterministic trends and random shocks. The deterministic trend reflects wage growth due to work experience and age, whereas the random shock captures the impact of macroeconomic environment changes and individual factors on wages. The labor income of an individual worker aged a in period t is expressed as follows:

(2) $$lnw_{a,t}^{i}=ln{\bar{w}}_{a}^{i}+\mu_{i}t+\rho_{a,t}^{i},a<x_{R}^{i}$$

Here, $i\in \left\{M,F\right\}$ represents the male (husband) and female (wife), respectively. The deterministic average wage for each age is denoted as ${\bar{w}}_{a}^{i}$. The drift term for labor income is $\mu_{i}$, reflecting the positive impact of technological progress and increased work experience on labor income. The random shock term is age- and time-dependent and is further expressed as $\rho_{a,t}^{i}=\varphi_{a}^{i}+\varepsilon_{t}^{i}$. Here, $\varphi_{a}^{i}$ represents the age-related persistent shock, defined as $\varphi_{a}^{i}=\vartheta^{i}\varphi_{a-1}^{i}+\upsilon_{a}^{i}$, where $\upsilon_{a}^{i}\sim N(0,{\sigma_{\upsilon }^{i}}^{2})$ and $\varphi_{x}^{i}=0$. $\varepsilon_{t}^{i}$ represents the time-related random shock and follows a normal distribution, $N(0,{\sigma_{\varepsilon }^{i}}^{2})$.

#### 3.1.2. Women’s Opportunity Costs

This study sets the opportunity cost of fertility as a lifelong impact on women’s wages (fertility penalty). Specifically, for each child a family has, the woman may experience an income shock, where $\pi_{k}$ represents the proportion of wage reduction due to the birth of the k-th child (considering policy constraints, this study assumes a maximum of three children). For simplicity, it is assumed that fertility penalties are independent of each other. Therefore, after giving birth to $n_{x+t}$ children in period t, the ratio of the woman’s wage to her previous wage is $z_{x+t}=\prod_{k=1}^{n_{x+t}}\left(1-\pi_{k}\right)(0\leq n_{x+t}\leq 3)$, with the convention that $\prod_{k=1}^{0}\left(1-\pi_{k}\right)=1$. Furthermore, based on the core tenet of loss aversion within prospect theory, individuals’ psychological sensitivity to losses is far greater than to equivalent gains (Kahneman & Tversky, 2013). This asymmetry leads to a disproportionate perception of the income losses incurred by childbearing; consequently, its inhibitory effect on household fertility decisions is highly likely to transcend the measurable economic income effects alone.

Considering maternity leave policies and the need for childcare during a child’s early years, this study assumes the following income process for women after childbirth. Women are entitled to α years of paid maternity leave, during which their wages remain consistent with pre-childbirth levels.

Therefore, for a female aged a in period t ($t\geq t_{k}$) who gives birth to the k-th child (k=1,2,3) in period $t_{k}$ (where $t_{k}\leq x_{F}-x$) and subsequently possesses n children (n=1,2,3), her wage level $w_{a,t}^{FF}\left(n\right)$ is defined as follows:

(3) $$w_{a,t}^{FF}\left(n\right)=\left\{\begin{array}{cc} z_{n-1}w_{a,t}^{F} & t_{k}\leq t\leq t_{k}+\alpha \\ z_{n}w_{a,t}^{F} & t>t_{k}+\alpha \end{array}\right.$$

where $w_{a,t}^{FF}\left(0\right)=w_{a,t}^{F}$.

#### 3.1.3. Post-Retirement Income Process

During retirement, household income comes from pensions. This study assumes that the pension amount is related to the pension replacement rate and the final wage, and pension income remains constant throughout the retirement period.

The pension income for an individual i (where $i\in \left\{M,F\right\}$) aged a is expressed as follows:

(4) $$w_{a,t}^{i}=\delta^{i}{\bar{w}}^{i},a\geq x_{R}^{i}$$

where $\delta^{i}$ represents the pension replacement rate for individual i. Here, ${\bar{w}}^{i}$ denotes the retirement wage of individual i with ${\bar{w}}^{M}=w_{x_{R}^{M}-1,x_{R}^{M}-1-x}^{M}$ for males and ${\bar{w}}^{F}=w_{x_{R}^{F}-1,x_{R}^{F}-1-x}^{FF}\left(n_{x_{R}^{F}-1}\right)$ for females, where $n_{x+t}$ further represents the number of children for an individual aged x+t.

In summary, the household’s wage (or pension) income in period t can be expressed as follows:

(5) $$w_{x+t,t}=w_{x+t,t}^{M}+w_{x+t,t}^{FF}\left(n_{x+t}\right),n_{x+t}=0,1,2,3$$

### 3.2. Child-Rearing Costs

In addition to opportunity costs, households face deterministic child-rearing expenditures encompassing childbirth, childcare, consumption, education, and housing. These costs are nearly unavoidable and exert significant constraints on fertility decisions due to their substantial financial burden, thus necessitating their explicit incorporation into the model. Specifically, the deterministic child-rearing costs include the following.

First, one-time costs associated with pregnancy and childbirth pe. These costs are fixed and occur exclusively during the childbearing period.

Second, the childcare cost for newborns L, which covers the caregiving expenses required before the child enters kindergarten $\left(t\leq t_{k}+2\right)$.^5^

Third, periodic child consumption costs parent. These include expenditures for raising children before adulthood and financial support after they reach adulthood. The expenditure amount varies with the child’s age and increases over time. The child consumption costs for a household during period t for a child aged a can be expressed as ${parent}_{t,a}$, which grows at a rate of $\mu_{parent}$ over time.

Fourth, periodic education costs edu. These expenditures also vary with the child’s age and increase gradually over time. We define the education expenditure for a household during period t for a child aged a as ${edu}_{t,a}$, which grows at a rate of $\mu_{edu}$ over time.

Fifth, marginal housing costs he. These costs arise from increased housing demand due to family size expansion after childbearing.^6^ We assume each additional child requires ∆H additional square meters of housing space. The housing demand is realized immediately upon childbirth, with all households adopting mortgage financing: a down payment is made at childbirth, and the remaining balance is repaid through equal installments comprising principal and interest until retirement age.^7^

For analytical tractability, this study assumes that the rigid child-rearing costs across children are mutually independent. The total household child-rearing cost equals the sum of individual costs across all children, i.e.,

(6) $$e_{x+t}=peI_{\left\{t=t_{k}\right\}}+LI_{\left\{t\leq t_{k}+2\right\}}+\sum_{k=1}^{n_{x+t}}{edu}_{t,a,k}+\sum_{k=1}^{n_{x+t}}{parent}_{t,a,k}+\sum_{k=1}^{n_{x+t}}{he}_{x+t}^{k}$$

Here, ${edu}_{t,a,k}$, ${parent}_{t,a,k}$, and ${he}_{x+t}^{k}$ denote the education costs, child consumption costs, and housing costs, respectively, in period t for the k-th child aged a. The number of children satisfies $n_{x+t}=0,1,2,3$.

### 3.3. Household Utility and Dynamic Programming Problem

#### 3.3.1. Household Utility

Following the utility function settings in (J. Zhang & Zhang, 2007) and (Yew & Zhang, 2013), this study assumes that the household utility in each period comes from three components: the first part is the total household consumption, which is the sum of the consumption of all family members during their lifetime. The second part is the utility generated by the number of children in the family during their lifetime. The third part is the bequest utility derived from the legacy left to the children after death.

For the utility during the lifetime, referring to (Billari et al., 2009) for the specific form of the utility function, the household utility function in period t is assumed to be $U_{x+t}=u\left(c_{x+t},n_{x+t}\right)$. However, because this study introduces rigid child-rearing costs, the family may face bankruptcy. When the family is not bankrupt, the utility function takes the form:

(7) $$u\left(c_{x+t},n_{x+t}\right)=\frac{c_{x+t}^{1-\gamma }-1}{1-\gamma }+I_{\left\{n_{x+t}\neq 0\right\}}\zeta \frac{n_{x+t}^{1-\kappa }}{1-\kappa }$$

Here, it is assumed that the family starts at age x, so in period t, the family’s age is x+t. The family’s consumption in period t is denoted by $c_{x+t}$, and the number of children in the family in period t is denoted by $n_{x+t}$. The subjective parameters embedded within the household utility function shape fertility decisions by capturing factors such as subjective perceptions and social expectations.^8^ Specifically, the parameter representing the preference for children (ζ) reflects the psychological impact of traditional fertility concepts and maternal role identity (Bertrand et al., 2015; Adda et al., 2017). The household’s coefficient of relative risk aversion regarding the number of children (κ) captures the classical “quantity–quality” trade-off faced when making fertility choices (Begall & Mills, 2011; Sommer, 2016). Furthermore, the coefficient of relative risk aversion (γ) regarding consumption mirrors the household’s degree of prudence when engaging in precautionary savings to smooth lifelong consumption (Chetty & Szeidl, 2007).

Currently, there are two main approaches in academia to handle family bankruptcy. The first approach maintains the form of the utility function in the case of bankruptcy but assumes that bankrupt families can receive a minimum living allowance provided by the government to ensure minimum consumption, as seen in Capatina (2015) and İmrohoroğlu and Zhao (2018). The second approach is grounded in prospect theory, assuming a structural shift in the utility function in the event of bankruptcy (or under insolvency) to reflect the loss aversion inherent in individual behavior, as exemplified by Levy (2016) and Pagel (2017). This study adopts the second approach to handle family bankruptcy, specifically by allowing negative consumption and introducing a penalty coefficient for negative consumption. This is due to the following. First, based on the psychological concept of loss aversion within prospect theory, individuals weigh perceived losses significantly more heavily than gains. This asymmetry implies that the anticipated losses associated with childbearing may exert an influence on decision-making that goes far beyond measurable income effects alone. The introduction of the negative consumption penalty coefficient is precisely designed to reflect the household’s aversion to a potential decline in living standards induced by childbirth. Second, the model established in this study is not an equilibrium model and does not consider the budget balance of government-provided minimum living allowances. Third, introducing a minimum living allowance would alter the family’s consumption, savings, and fertility decisions (because bankrupt families would not incur costs to obtain the minimum living allowance). In summary, this study combines prospect theory to make the following assumption about the form of the utility function in the case of family bankruptcy:

(8) $$u\left(c_{x+t},n_{x+t}\right)=-\lambda \frac{{\left(-{cr}_{x+t}\right)}^{1-\gamma }-1}{1-\gamma }+I_{\left\{n_{x+t}\neq 0\right\}}\zeta \frac{n_{x+t}^{1-\kappa }}{1-\kappa }$$

where ${cr}_{x+t}$ represents the family’s negative assets in period t (calculated as the principal and interest of the family’s savings from the previous period plus current income minus the rigidly paid child-rearing costs). The parameter λ represents the penalty coefficient for negative consumption. This parameter reflects the loss aversion psychology embedded within prospect theory, implying that households are strongly averse to a “cliff-edge” drop in their standard of living or being burdened with unpayable debt.

Finally, when the parents in the family pass away, the remaining assets become a legacy, and the bequest utility function is expressed as follows:

(9) $$u_{2}\left(A\right)=\xi \frac{A^{1-\gamma }-1}{1-\gamma }$$

where A represents the bequest, which is the sum of the principal and interest in the final period, and the bequest motive is denoted by ξ. From the perspective of behavioral economics, it reflects the subjective psychological weight that parents assign to intergenerational wealth transfer out of intertemporal altruism (Andreoni, 1989; Kopczuk & Lupton, 2007, Barro & Becker, 1989).

#### 3.3.2. Dynamic Programming Process

The family maximizes its lifetime expected utility through consumption, savings, and fertility decisions in each period. The decision-making process follows the following assumptions. First, before reaching the maximum fertility age $x_{F}$, the family decides in each period whether to have a child. Once the family exceeds $x_{F}$, it no longer makes fertility decisions. Second, the family can have only one child per period, and each child provides the same utility. Third, the family decides whether to have a child based on the current state, rather than planning all fertility decisions at the outset. Fourth, the household decisions are based on lifetime expected utility, and this study assumes that the emotional factors related to children are already included in the utility derived from having children. The dynamic programming process for the family’s consumption, savings, and fertility decisions is as follows.

When $0\leq t\leq x_{F}-x$, the family is in the fertility period, and the Bellman equation for the family’s decision is expressed as follows:

(10) $$V_{x+t}\left(s_{x+t-1},z_{x+t},n_{x+t},{\overset{⃑}{y}}_{x+t}\right)=\underset{c_{x+t},s_{x+t},n_{x+t+1}1_{\left\{n_{x+t}<3\right\}}}{max}\left\{\begin{array}{l} u\left(c_{x+t},n_{x+t}\right)\\ +\beta I_{\left\{n_{x+t+1}=n_{x+t}\right\}}V_{x+t+1}\left(s_{x+t},z_{x+t+1}=z_{x+t},n_{x+t+1},{\overset{⃑}{y}}_{x+t+1}\right)\\ +\beta I_{\left\{n_{x+t+1}=n_{x+t}+1\right\}}EV_{x+t+1}\left(s_{x+t},z_{x+t+1},n_{x+t+1},{\overset{⃑}{y}}_{x+t+1}\right) \end{array}\right\}$$

where $V_{x+t}$ represents the family’s lifetime expected utility in period t. $V_{x+t}$ includes four state variables: $s_{x+t-1}$, $z_{x+t}$, $n_{x+t}$, and ${\overset{⃑}{y}}_{x+t}$. $s_{x+t-1}$ denotes the family’s savings from the previous period, $z_{x+t}$ represents the wage state of the woman due to fertility penalties, $n_{x+t}$ indicates the current number of children in the family, and ${\overset{⃑}{y}}_{x+t}$ represents the age vector of the children.

During the fertility period, the family’s budget constraint is expressed as follows:

(11) $$s_{x+t-1}\left(1+r\right)+w_{x+t,t}=c_{x+t}+s_{x+t}+e_{x+t}$$

where r is the interest rate on family savings.

When $x_{F}-x<t<x_{T}-x$, the family enters the non-fertility period, during which it only makes consumption and savings decisions. The Bellman equation for this period is expressed as follows:

(12) $$\begin{array}{l} V_{x+t}\left(s_{x+t-1},z_{x+t},n_{x+t},{\overset{⃑}{y}}_{x+t}\right)\\ \;\;\;\;\;\;\;\;\;\;\;\;=\underset{c_{x+t},s_{x+t}}{max}\left\{u\left(c_{x+t},n_{x+t}\right)+\beta V_{x+t+1}\left(s_{x+t},z_{x+t+1},n_{x+t+1},{\overset{⃑}{y}}_{t+1}\right)\right\} \end{array}$$

During this period, fertility decisions are no longer made, and there are no fixed expenses related to childbirth. The family’s budget constraint is expressed as follows:

(13) $$s_{x+t-1}\left(1+r\right)+w_{x+t,t}=c_{x+t}+s_{x+t}+e_{x+t}$$

When $t=x_{T}-x$, the household enters the final period of its lifecycle, no longer considering future expected utility but solely focusing on posthumous bequest utility. At this stage, the household integrates savings, post-fertility wage status, number of children, and their ages, making consumption and bequest decisions under budget constraints to ultimately maximize the total utility of current consumption and bequests.

(14) $$V_{x+t}\left(s_{x+t-1},z_{x+t},n_{x+t},{\overset{⃑}{y}}_{x+t}|n_{x},{\overset{⃑}{y}}_{x}\right)=\underset{c_{x+t},s_{x+t}}{max}E\left\{u\left(c_{x+t},n_{x+t}\right)+u_{2}\left(A\right)\right\}$$

The budget constraint at this point is expressed as follows:

(15) $$s_{x+t-1}\left(1+r\right)+w_{x+t,t}=c_{x+t}+s_{x+t}+e_{x+t}$$

## 4. Parameter Calibration

Given the complexity of the aforementioned model, which precludes deriving closed-form solutions, this study employs numerical methods for computation. This section first calibrates parameters to align the numerical solutions with empirical realities, whereas the subsequent section utilizes the calibrated model for analytical exploration and policy effect simulations.

This study draws upon the parameter calibration methodologies of DellaVigna (2018), Gill and Prowse (2012), and Laibson et al. (2026), employing structural estimation to calibrate the model parameters.^9^ Specifically, the parameters in this paper can be divided into two categories: objective parameters and subjective parameters. Objective parameters are estimated based on actual data and include parameters related to the family wage process $\left\{w_{x+t,t}\right\}$: $\left\{\mu_{i},\rho_{a,t}^{i},\delta^{i}\right\}$, family savings interest rate r, fixed costs during childbirth pe in rigid child-rearing costs, childcare costs for newborns L, childcare costs ${parent}_{t,a}$ and their growth rate $\mu_{parent}$, educational costs ${edu}_{t,a}$ and their growth rate $\mu_{edu}$, and family housing costs ${he}_{x+t}^{i}$. Subjective parameters are estimated using the simulated method of moments and include the relative risk aversion coefficient for consumption γ, the relative risk aversion coefficient for the number of children κ, the preference for children ζ, the penalty coefficient for negative consumption in case of bankruptcy λ, and the bequest motive ξ.

This study first makes assumptions about demographic characteristics. According to data published in the China Population Census Yearbook 2020, the average age at first marriage in China has been increasing over the decade from 2010 to 2020, increasing from 24.89 years in 2010 to 28.67 years in 2020. For ease of subsequent adjustments, this study initially assumes that the family’s starting age is x=25, the maximum age is $x_{T}=90$, the wife’s maximum fertility age is $x_{F}=49$, the husband’s (male) retirement age is $x_{R}^{M}=60$, and the wife’s (female) retirement age is $x_{R}^{F}=55$.

### 4.1. Calibration of Income Process and Women’s Opportunity Cost Parameters

#### 4.1.1. Wage Process and Pension Replacement Rate

The estimation of wages and pension replacement rates proceeds as follows. First, following Cho et al. (2021), this study estimates the age profiles of average wages for males and females. Second, drawing on Heathcote et al. (2010) and Tonetti (2011), the wage growth rates for men and women $\left\{\mu_{M},\mu_{F}\right\}$ are estimated using the minimum distance method. Third, following Heathcote et al. (2010), Tonetti (2011), and Tauchen (1986), the stochastic component of wages is estimated and discretized into five states. Finally, pension replacement rates are calibrated based on current pension policies. For brevity, the detailed calibration procedures are presented in “Appendix A.1 Calibration of the Wage Process and Pension Replacement Rates.”

#### 4.1.2. Estimation of the Fertility Penalty

Given the limited availability of 2022 third-child data, this study calibrates fertility penalty parameters based on first- and second-child data. Using CFPS 2020 and 2022 data, we selected female samples who had no children in 2020 but gave birth to their first child in 2022, comparing their actual wages to their counterfactual wages (theoretical wages without childbirth) to derive sample values for $1-\pi_{1}$. Similarly, for samples who already had one child in 2020 and gave birth to a second child in 2022, we compared their actual wages to counterfactual wages (theoretical wages without a second child) to obtain sample values for $1-\pi_{2}$.

This study first employs the Kolmogorov–Smirnov (K-S) test to examine whether $\pi_{1}$ and $\pi_{2}$ follow identical distributions. The test yields a K-S statistic of KS=0.134847 and a *p*-value of 0.4426. At a significance level of α=0.05, we fail to reject the null hypothesis of distributional equivalence, thus concluding that the fertility penalties associated with first and second childbirths are statistically indistinguishable in their distributional properties.

Subsequently, this study combines the fertility penalty samples from first and second childbirths and performs distribution fitting. The generalized Pareto distribution $G\left(x,-0.2455,0.4063\right)$, which exhibits the best fit, is selected to characterize the distribution of fertility penalties π. Following Klugman et al. (2012), the distribution of the fertility penalty is discretized, and the results are reported in Table 5.

It is necessary to point out that, due to the loss aversion psychology within prospect theory (Kahneman & Tversky, 2013), individuals weigh perceived losses more heavily than gains. Consequently, the actual impact of the motherhood penalty on fertility intentions is highly likely to transcend the measurable economic income effects alone.

### 4.2. Calibration of Parameters Related to Child-Rearing Costs

#### 4.2.1. Fixed Costs During Pregnancy

Pregnancy-related fixed expenditure refers to the series of costs incurred during the childbearing period, including pre-pregnancy preparation, childbirth, and postpartum recovery. Based on estimates from the China Fertility Cost Report 2024, this study sets pre-pregnancy expenditures at approximately 10,000 CNY (covering expenses such as medical registration, nutritional supplements, and documentation), delivery costs at 15,000 CNY (including hospitalization and delivery fees), and postpartum recovery costs at 7000 CNY. Thus, the total fixed expenditure during the childbearing period amounts to 32,000 CNY, denoted as pe=32,000.

#### 4.2.2. Childcare Costs for Newborns

Childcare costs for newborns refer to the expenditures incurred for children aged 0–2 who require full-time care. In the Chinese context, newborn care is typically provided through three channels: care by grandparents, hired maternity nannies, or maternal career interruption. As this study focuses on two-generation households, grandparental care is excluded. According to current conditions in China’s childcare market, whether the family relies on maternal caregiving (which requires the mother to temporarily exit the labor market) or hires a maternity nanny (allowing the mother to continue working), the economic burden borne by the household is broadly comparable.^10^ Based on this consideration, this study sets the childcare cost for newborns (ages 0–2) equal to the mother’s wage income for the 3 years following childbirth.^11^

#### 4.2.3. Child Consumption Costs

This study divides child development into three distinct stages. The first stage spans ages 0 to 2 years, during which a child’s required dietary structure and caregiving costs differ significantly from other periods, and the child has not yet reached school age. The second stage covers ages 3 to 22 years, a period where the child’s dietary requirements largely converge with those of adults. The final stage begins after age 22 years, when children have typically graduated from college and achieved financial independence. Although parents may still provide inter vivos transfers during this period, these transfers are no longer non-discretionary expenditures, thereby justifying separate consideration. Based on the CFPS survey data, we calibrate the specific values of child-rearing expenditures for each stage. The calibration outcomes are reported in Table 6, and the detailed calibration methodology is provided in Appendix A.2, “Child Consumption Costs.”

#### 4.2.4. Education Costs

Similarly to the wage process, this study employs polynomial fitting to estimate child education expenditures. Using the “total education expenditures in the past 12 months (RMB)” field from the CFPS 2022 dataset as our data source, we adopt a fourth-order polynomial fitting to characterize the dynamics of education expenditures across ages. Subsequently, by comparing the three waves of survey data from CFPS 2018, CFPS 2020, and CFPS 2022, we utilize the minimum distance method to estimate the growth rate of education expenditures over time. The estimation results are reported in Table 7, and the detailed calibration methodology is provided in Appendix A.3, “Education Costs.”

#### 4.2.5. Housing Costs of Childbirth

This study identifies the marginal housing demand associated with childbearing using a regression-based approach. Taking household housing area as the dependent variable and household size as the core explanatory variable, and controlling for income, age, debt status, and health status, the regression results show a significant positive correlation between housing area and household size, with a coefficient of 13.076. Based on this finding, this study assumes that each additional family member increases housing demand by approximately 13.1 square meters. Consistent with China’s housing loan policies, the down payment ratio is set at χ=30%, and the mortgage interest rate at $r_{m}=3.5\%$.

Subsequently, following Hu (2022) and Fu et al. (2021), an ARIMA (3, 1, 3) model is employed to forecast the future housing price trajectory, which is then used to infer the housing price faced by the household when giving birth to the k-th child. Finally, combining the down payment, loan repayment horizon, and the forecast housing price, the housing cost associated with the k-th child in period $t_{k}$, denoted as $he_{x+t}^{k}$, is determined. The detailed formula and parameter settings are provided in “Appendix A.4. Calibration of Housing Costs.”

### 4.3. Structural Estimation of Subjective Parameters and Model Baseline Results

To incorporate subjective psychological factors into our quantitative mathematical model, this study follows the methodologies of DellaVigna (2018), Gill and Prowse (2012), and Laibson et al. (2026), employing the Simulated Method of Moments (SMM) to calibrate household subjective parameters. During the calibration process, the number of children per household is selected as the target moment, thereby aligning the model’s simulated outcomes with actual empirical fertility behaviors. The calibration proceeds in the following steps. First, a set of subjective household parameters $\Theta =\left\{\gamma ,\beta ,\kappa ,\zeta ,\xi ,\lambda \right\}$ is conjectured. Second, 100,000 household life-cycle paths are generated using Monte Carlo simulation. Third, the fertility decisions along each simulated path are computed, and the average number of children per household is obtained. Finally, the discrepancy between the model-implied results and the empirical fertility data, denoted as $g_{x+t}(\Theta )$, is calculated. This procedure is repeated iteratively until the difference becomes sufficiently small. For brevity, the detailed formulas and parameter settings used in the simulated method of moments are provided in “Appendix A.5. Calibration of Household Subjective Parameters.”

All calibrated parameter values are summarized in Table 8.

**Figure 1:**
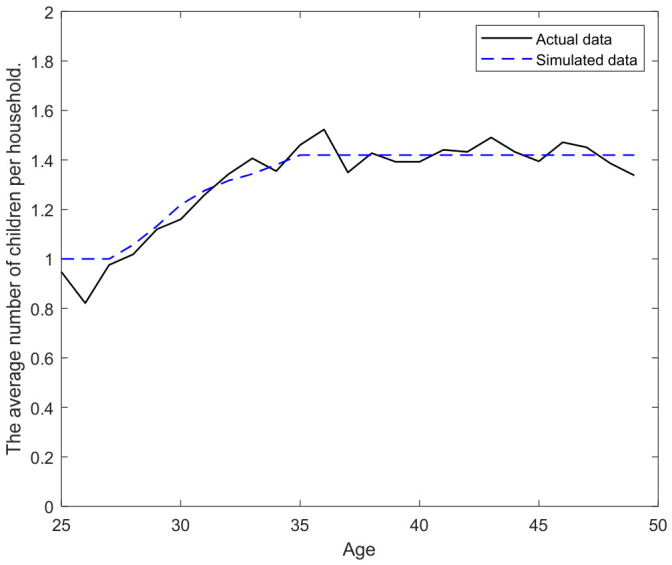
Fertility fitting by household age.

The model fitting results are presented in Figure 1. Comparison with actual data shows that, except for the initial periods, the model simulates fertility decisions reasonably well. The deviations in the early periods arise from three main factors. First, from a parameter calibration perspective, the timing of initial fertility decisions is uncertain, as real-world household formation does not uniformly begin at age 25. This inherent variability leads to unavoidable discrepancies during the calibration process. Second, computational limitations force all simulated households to start from identical initial conditions, resulting in uniform initial decisions (e.g., zero or one child). To improve model fit, the starting point is calibrated to one child.

## 5. Results and Analysis

Building on the theoretical model, this study conducts a series of simulation analyses to examine how various factors and policy interventions influence household fertility intentions.

### 5.1. The Impact of Women’s Opportunity Costs on Household Fertility Intentions

#### 5.1.1. The Impact of Fertility Penalty Levels on Household Fertility Intentions

A substantial body of research shows that women may experience a 5–30% loss in earnings due to childbirth (Kleven et al., 2019; Budig & England, 2001; Jia & Dong, 2013; Gangl & Ziefle, 2009), a decline in occupational status (Abendroth et al., 2014), and even career interruptions (Polachek, 1985; Drasch, 2013). Drawing on these findings, this study conducts simulation analyses based on the baseline model by varying the magnitude of the fertility penalty while holding the probability of penalty occurrence constant to examine how different levels of fertility penalties affect household fertility intentions.

Scenario 1: No fertility penalty.

Scenario 2: Fertility penalty set at 50% of the baseline model, meaning that the probabilities are adjusted to: 0.27 for no wage reduction, 0.58 for a 9.16% wage reduction, and 0.15 for a 37.39% wage reduction.

Scenario 3: Fertility penalty set at 150% of the baseline model, corresponding to 0.27 for no wage reduction, 0.58 for a 27.48% wage reduction, and 0.15 for a 100% wage reduction.^12^

**Figure 2:**
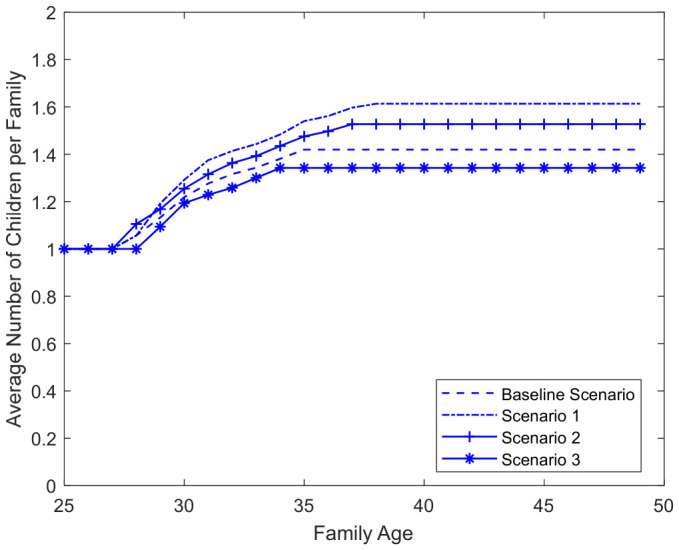
Comparison of fertility intentions under different levels of fertility penalties.

The comparative results of household fertility intentions under these different levels of fertility penalties are presented in Figure 2 and Table 9.

Figure 2 and Table 9 demonstrate that greater fertility penalties correlate with lower household fertility intentions, earlier termination of childbearing decisions, and shorter fertility time windows. Compared to the baseline scenario, eliminating fertility penalties increases the proportion of two-child families to 61.36% and delays the childbearing termination age by 3 years. Conversely, under the 150% fertility penalty scenario, the proportion of two-child families drops to 34.23%, with the termination age occurring 1 year earlier. The theoretical model confirms that larger fertility penalties amplify wage losses for women due to childbearing, thereby raising the implicit costs of reproduction. Consequently, reducing fertility penalties effectively enhances household fertility intentions.

#### 5.1.2. The Impact of Career Interruption Duration on Household Fertility Intentions

In real labor markets, women may continue working after childbirth or may temporarily or permanently exit the labor force (Kluve & Tamm, 2013; Picchio et al., 2021; Bhalotra et al., 2023). Based on this observation, and building upon the baseline model, this study incorporates and adjusts the duration of career interruption to further examine how different lengths of labor market exit influence household fertility intentions. Here, career interruption refers to the period during which women temporarily withdraw from the labor market due to childbirth.

Scenario 4: Women temporarily exit the labor market after childbirth, with a career interruption of 4 years.

Scenario 5: Women temporarily exit the labor market after childbirth, with a career interruption of 5 years.

Scenario 6: Women permanently exit the labor market after childbirth.

**Figure 3:**
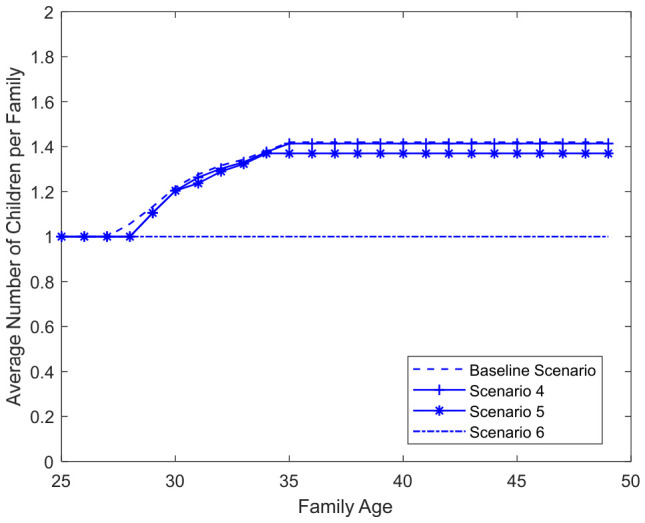
Comparison of fertility intentions under different career interruption durations.

The comparative results of household fertility intentions under different durations of career interruption are presented in Figure 3 and Table 10.

Figure 3 and Table 10 indicate that career interruption due to childbirth reduces household fertility intentions, and longer interruptions are associated with lower fertility. Under the baseline scenario and the scenarios with 4-year and 5-year career interruptions, the average number of children per household at age 49 is 1.4196, 1.4137, and 1.3703, respectively. When women permanently exit the labor market after childbirth, households choose to have only one child and no longer consider having a second child.^13^ This result is intuitive. Specifically, the longer the career interruption, the greater the reduction in household income and the weaker the resulting fertility intention.

### 5.2. The Impact of Household Income and Child-Rearing Costs on Household Fertility Intentions

Household economic conditions constitute an important determinant of fertility decisions (Leibenstein, 1957; Becker & Tomes, 1976). In recent years, China has attached great importance to addressing low fertility rates and has identified reducing childrearing costs, such as through childcare subsidy programs, as a key policy tool for increasing fertility intentions. Based on this context, and building upon the baseline model, this study conducts simulation analyses by adjusting household income and childrearing costs to further examine how these factors influence fertility intentions.^14^

Scenario 7: Household income and childrearing costs are both set to 70% of the baseline values.

Scenario 8: Household income is increased to 150% of the baseline, while childrearing costs remain unchanged.

Scenario 9: Household income and childrearing costs are both increased to 150% of the baseline values.

**Figure 4:**
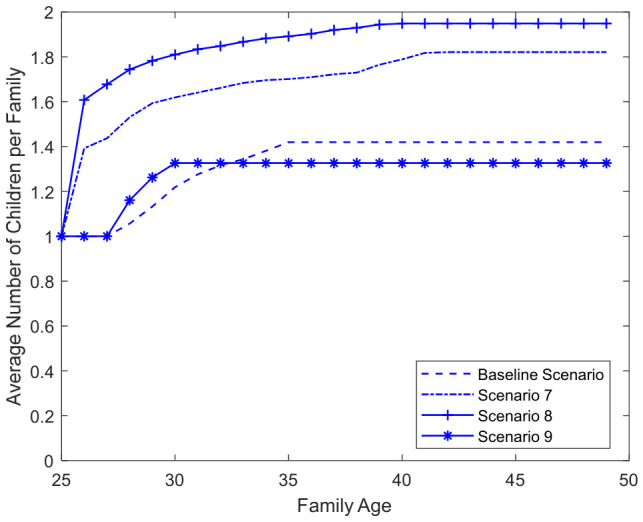
Comparison of fertility intentions under different income and childrearing costs.

The comparative results of household fertility intentions under these different income–cost scenarios are presented in Figure 4 and Table 11.

Figure 4 and Table 11 show that household fertility intentions increase with higher income but decline with higher childrearing costs, and that variations in childrearing costs exert a stronger influence on fertility decisions. Specifically, when both income and childrearing costs are reduced to 70% of the baseline model, the proportion of two-child households rises to 82.13%, and the age at which families stop considering further childbearing is delayed by 7 years. When income is increased to 150% of the baseline while keeping childrearing costs unchanged, the proportion of two-child households increases to 94.89%, with the stopping age postponed by 5 years. However, when both income and childrearing costs are increased to 150% of the baseline, the proportion of two-child households declines to 32.64%, and the stopping age occurs 5 years earlier. These results mirror real-world patterns, in which households in more developed regions face higher incomes and higher childrearing costs, yet exhibit lower fertility intentions.

### 5.3. The Impact of Different Years of Schooling on Household Fertility Intentions

Years of education constitute an important determinant of human capital accumulation and the timing of fertility. The existing literature (Willis, 1973; Balbo et al., 2013; Hanappi et al., 2017) indicates that higher educational attainment not only increases human capital and future labor income but also increases the opportunity costs of childbearing, making women more inclined to postpone fertility or adopt more cautious fertility strategies. In China, the age pattern of fertility similarly exhibits pronounced educational stratification, with highly educated groups tending to marry and have children at later ages. Drawing on China’s typical educational stratification and income gradients in the labor market, and following Griliches and Mason (1972), this study assumes that each additional year of schooling increases wage income by approximately 5%. Accordingly, building upon the baseline model, this study adjusts the household’s initial age and income level to further examine how different lengths of schooling affect fertility intentions.

Scenario 10: The household begins fertility consideration at age 27, with income set to 110% of the baseline level.

Scenario 11: The household begins fertility consideration at age 29, with income set to 120% of the baseline level.

Scenario 12: The household begins fertility consideration at age 31, with income set to 130% of the baseline level.

**Figure 5:**
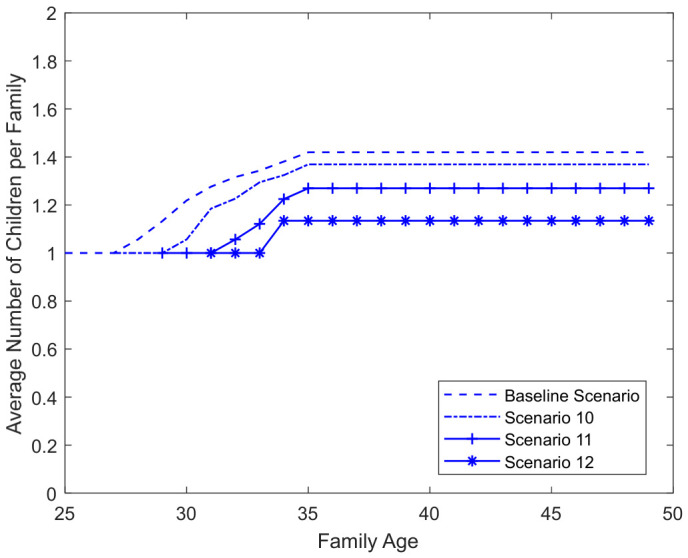
Comparison of fertility intentions under different education years.

The comparative results of household fertility intentions under different levels of schooling (reflected by differences in initial age and income) are presented in Figure 5 and Table 12.

Figure 5 and Table 12 show that the model captures real-world patterns well: households with longer years of education exhibit lower fertility intentions. More years of schooling delay the initiation of fertility decision-making and, at the same time, increase household income. Generally, later initiation of fertility decisions reduces fertility intentions, whereas higher income tends to increase them. Figure 5 and Table 12 indicate that, relative to the income effect, the negative impact of delayed fertility decision-making is more pronounced. Specifically, compared with the baseline scenario in which fertility decisions begin at age 25, delaying the start by 2, 4, and 6 years reduces the average number of children per household at age 49 by 0.0504, 0.1498, and 0.2852, respectively.

### 5.4. The Impact of Fertility Subsidies on Household Fertility Intentions

Low fertility has become a global demographic norm in the 21st century, and fertility-support policies represent an important attempt to raise fertility rates. Countries around the world have actively explored and implemented various forms of economic support tailored to their national contexts. For example, in 2023, Russia provided a one-time subsidy of more than 580,000 rubles (approximately 51,000 CNY) for families with a first child.^15^ Accordingly, this study sets Scenario 13, offering a one-time childbirth subsidy of 50,000 CNY. In France, families with children under age three receive a basic allowance of approximately 185 euros per month (about 18,000 CNY per year).^16^ Based on this policy, Scenario 14 provides an annual subsidy of 20,000 CNY for children aged 0–2. Furthermore, beginning in 2025, France grants families with at least two children under age 18 an allowance of 142.5–285 euros per month (14,000–28,000 CNY per year), equivalent to roughly 7000–14,000 CNY per child annually. Drawing on this, Scenario 15 provides an annual subsidy of 5000 CNY for each child aged 0–22.

Based on these policy examples, this study constructs three types of fertility subsidies to simulate the effects of alternative policy designs on household fertility intentions.

Scenario 13: A one-time childbirth subsidy of 50,000 CNY.

Scenario 14: An annual subsidy of 20,000 CNY for children aged 0–2.

Scenario 15: An annual subsidy of 5000 CNY for children aged 0–22.

**Figure 6:**
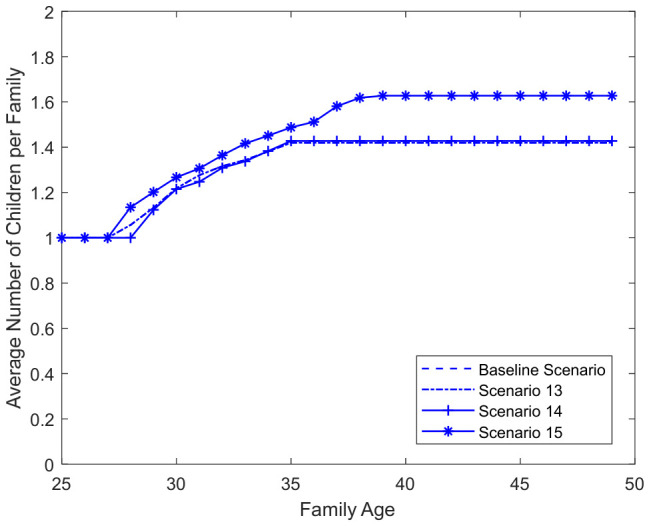
Comparison of different subsidy forms.

By comparing these three policy scenarios, the model systematically assesses the differential effects of short-term shocks (one-time subsidy), medium-term support (focusing on early childcare years), and long-term benefits (covering the entire pre-adult period). This provides theoretical foundations and quantitative simulation evidence for developing a more precise and structurally optimized fertility support system. The comparative results under different fertility subsidy policies are presented in Figure 6 and Table 13.

Figure 6 and Table 13 indicate that continuous fertility subsidies have a more substantial effect on increasing fertility intentions. Specifically, compared with the baseline scenario, providing an annual subsidy of 5000 CNY for children aged 0–22 increases the average number of children per household at age 49 by 0.2079 and delays the age at which families stop childbearing by 4 years. Under the scenario of an annual subsidy of 20,000 CNY for children aged 0–2, the average number of children increases by 0.0080, while the stopping age remains unchanged. In contrast, providing a one-time subsidy of 50,000 CNY at childbirth does not alter either the average number of children at age 49 or the stopping age.

### 5.5. The Impact of Childcare Policies on Household Fertility Intentions

Childcare services play a crucial role in reducing the risk of maternal career interruption and alleviating the time and opportunity costs of childrearing (C. Zhang & Managi, 2021; Oehrli et al., 2024). In June 2025, China’s National Health Commission, together with the National Development and Reform Commission and five other ministries, jointly issued the Opinions on Accelerating the Development of an Inclusive Childcare Service System, which explicitly called for “strengthening the construction of comprehensive childcare service centers.” To simulate the effects of different childcare policies on household fertility intentions, this study refers to the fee range of public kindergarten childcare and accommodation charges in Beijing (550–1050 CNY per month) and extends the analysis to include a free childcare scheme to explore the upper bound of policy support.^17^ Accordingly, three childcare cost scenarios are constructed:

Scenario 16: Annual childcare cost of 6600 CNY for ages 0–2.

Scenario 17: Annual childcare cost of 12,600 CNY for ages 0–2.

Scenario 18: Free childcare for ages 0–2.

**Figure 7:**
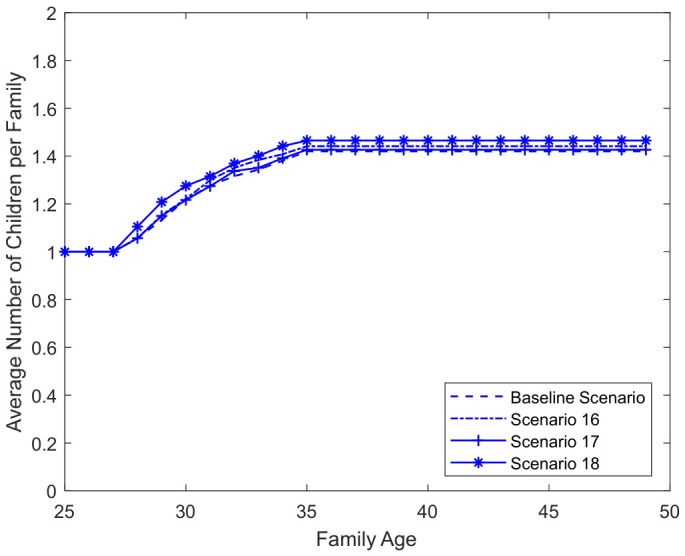
Comparison of effects under different childcare costs.

These three policy scenarios correspond to varying levels of fiscal investment and public support within the childcare service system. They aim to compare the marginal impact of reduced childcare costs on maternal opportunity costs, employment continuity, and fertility intentions, thereby revealing the potential incentive effects of childcare policies on household fertility decisions. The comparative results under different childcare policy scenarios are presented in Figure 7 and Table 14.

Figure 7 and Table 14 show that free childcare services have a more substantial effect on increasing fertility intentions. Specifically, compared with the baseline scenario, providing free childcare increases the average number of children per household at age 49 by 0.0454. Under the scenario with an annual childcare cost of 12,600 CNY, the average number of children increases by 0.0064, whereas an annual childcare cost of 6600 CNY increases the average number of children by 0.0219.

## 6. Conclusions

Against the backdrop of persistently declining fertility rates in China, examining the impact of women’s opportunity costs on fertility intentions is of considerable importance. Utilizing the CFPS 2022 dataset, this study first constructed an empirical model to explore the association between women’s opportunity cost and household fertility choices. The empirical results demonstrate that women’s opportunity cost exhibits a robust negative correlation with the actual number of children in a household. Building on the empirical analysis, this study incorporated women’s opportunity costs from childbearing and the rigid childrearing expenditures into a life-cycle model, and quantitatively evaluated the magnitude of their impact on household fertility decisions.

Subsequently, based on the baseline model, a series of simulation scenarios were developed to reveal how different factors influence household fertility intentions. The analysis yields several key findings, which are detailed below.

First, higher fertility penalties and longer career interruptions due to childbirth lower household fertility intentions. This result is highly consistent with existing empirical research evidences the heterogeneous effects across scenarios in which women continue working after childbirth, experience varying degrees of fertility penalties, or temporarily or permanently exit the labor market. These findings quantify the impact of potential fertility penalties faced by women in China’s labor market on fertility intentions.

Second, higher household income increases fertility intentions, whereas higher childrearing costs reduce them. In addition, the negative effect of rising childrearing costs is more pronounced. This aligns with the empirical findings that urban households and higher-income households tend to have fewer children. Under intense competition for education and childcare resources, the economic and psychological pressures associated with high-investment childrearing substantially suppress fertility intentions. This helps to explain the low fertility levels observed in highly developed cities such as Beijing and Shanghai.

Third, households with more years of schooling exhibit lower fertility intentions, consistent with the empirical results. Given China’s pronounced educational stratification and income gradients, as well as the accumulation of resources accompanying higher education, highly educated women face higher opportunity costs and tend to postpone childbirth until career and financial conditions stabilize, ultimately resulting in lower fertility intentions.

Fourth, the policy simulation results indicate that continuous fertility subsidies and free childcare services significantly enhance fertility intentions. This finding aligns with China’s ongoing policy direction of expanding childcare subsidies and building an inclusive childcare service system.

Building upon these findings, this study proposes the following policy recommendations.

The first recommendation is further improving a fertility-friendly labor market environment. The findings in this paper suggest that when women face higher opportunity costs of childbearing, households tend to exhibit lower fertility levels. Therefore, policy considerations could focus on further enhancing employment security and career development support mechanisms for women post-childbirth. Examples include exploring more flexible working arrangements, improving re-employment support systems, and optimizing fertility-inclusive mechanisms in career promotions, thereby mitigating the career disruption pressures that women experience due to childbearing, to some extent.

The second recommendation is gradually optimizing the socialized childcare support system. The model simulation results indicate that rising child-rearing costs substantially lower household fertility intentions. Consequently, developing inclusive and affordable childcare services and reducing the financial burden of household child-rearing expenditures may help to alleviate the constraints that high upbringing costs impose on household fertility choices. Concurrently, it is also essential to promote a more equitable distribution of childcare responsibilities within the household to relieve the time and labor pressures borne by women in child-rearing.

The third recommendation is further strengthening fertility support and social security mechanisms. The results of this study demonstrate that long-term income loss and career disruptions represent critical factors shaping household fertility choices. Hence, tailored to localized contexts, further exploring comprehensive fertility support policies for childbearing-age households, such as tax incentives, child allowances, and maternity insurance schemes, could mitigate the financial pressures that households face due to childbearing, to a certain extent.

It is necessary to clarify that the policy implications presented in this paper are primarily derived from correlational empirical results and mechanisms simulated within a lifecycle framework. Thus, the conclusions should be interpreted as potential directional insights rather than strict, causal policy evaluations. Furthermore, this study does not explicitly account for the fiscal costs, general equilibrium effects, or long-term policy efficiency associated with policy implementation; these dimensions remain vital avenues for deeper investigation in future research. Finally, endogenizing information acquisition channels and idiosyncratic household traits within the theoretical framework represents another vital and promising avenue for future research.

## Figures and Tables

**Table 1:** Variable definitions and descriptive statistics.

Variable Category	Variable	Definition	Mean	Std. Dev.	Min	Max
Dependent Variable	Actual Number of Children	Matched based on children’s information and counted for each family.	1.619	0.709	1	7
Core Explanatory Variable	Women’s Opportunity Costs	This equals 1 if the woman left work due to fertility or other family reasons; otherwise, it is 0.	0.051	0.219	0	1
Control Variables						
Female Characteristics	Age	Values	37.544	6.436	20	49
	Years of Education	Assigned based on educational level (see note below).	11.274	4.153	0	19
	Health Status	This equals 1 if healthy; otherwise, it is 0.	0.067	0.250	0	1
Spouse Characteristics	Spouse’s Years of Education	Assigned based on educational level (see note below).	11.505	3.790	0	22
	Spouse’s Health Status	This equals 1 if healthy; otherwise, it is 0.	0.064	0.244	0	1
Household Characteristics	Per Capita Household Income	In CNY, logarithmic transformation applied.	10.211	0.679	7.314	13.071
	Government Subsidy	This is 1 if receiving government subsidy; otherwise, it is 0.	0.262	0.440	0	1
	Debt	This is 1 if the household has debt; otherwise, it is 0.	0.484	0.500	0	1
Regional Characteristics	Urban–Rural Division	This is 1 for rural areas; otherwise, it is 0 for urban areas.	0.677	0.468	0	1
	Per Capita GDP	In 100 million CNY, logarithmic transformation applied.	11.235	0.376	10.714	12.156

Note: “Years of Education” is assigned based on educational level, divided into 8 categories: illiterate/no schooling = 0, primary school = 6, junior high school = 9, senior high school = 12, college = 15, bachelor’s degree = 16, master’s degree = 19, and doctoral degree = 22. Per capita household income is calculated based on the parents’ income and the total number of household members, including parents and children.

**Table 2:** Baseline regression results.

	Coefficient	Standard Errors
Women’s Opportunity Cost	−0.1547 **	0.0758
Age	0.0032	0.0029
Years of Education	−0.0207 ***	0.0074
Health Status	0.1864 *	0.1009
Spouse’s Years of Education	−0.0021	0.0071
Spouse’s Health Status	0.0382	0.0751
Per Capita Household Income	−0.3056 ***	0.0424
Government Subsidy	0.0232	0.0440
Debt	0.0240	0.0353
Urban–Rural Division	−0.1264 ***	0.0426
Per Capita GDP	0.1022	0.1965
Province Fixed	yes	
Constant	3.6901	2.2511
Observations	1243	
R2	0.3144	

Note: *, **, and *** indicate significance at the 10%, 5%, and 1% levels, respectively.

**Table 3:** Robustness test results.

	(1)	(2)	(3)	(4)	(5)	(6)	(7)
	Narrowed Observations	Ordered Probit	Ordered Logit	Poisson Regression	Adding Additional Control Variables	Alternative Explanatory Variable—Duration of Career Interruption	Alternative Explanatory Variable— Potential Wage Penalty
Women’s Opportunity Costs	−0.1534 ** (0.0776)	−0.3111 * (0.1711)	−0.5395 * (0.3189)	−0.0952 ** (0.0472)	−0.1382 * (0.0756)	−0.2263 ** (0.0872)	−0.0324 * (0.0186)
Control Variables	Yes	Yes	Yes	Yes	Yes	Yes	Yes
Province Fixed	Yes	Yes	Yes	Yes	Yes	Yes	Yes
Observations	1141	1243	1243	1243	1243	1196	1243
R2/Pseudo R2	0.3183	0.2059	0.2051	0.0370	0.3183	0.3190	0.3157

Note: Robust standard errors are reported in parentheses. * and ** indicate significance at the 10% and 5% levels, respectively.

**Table 4:** Heterogeneity analysis results.

	Urban–Rural Heterogeneity		Educational Attainment Heterogeneity	
	(1)	(2)	(3)	(4)
	Urban	Rural	Higher Education Attainment	Lower Education Attainment
Women’s Opportunity Costs	−0.1843 ** (0.0910)	−0.1206 (0.1610)	−0.2157 ** (0.0880)	−0.1254 (0.1326)
Control Variables	Yes	Yes	Yes	Yes
Province Fixed	Yes	Yes	Yes	Yes
Observations	842	401	702	589
R2	0.2756	0.3550	0.2126	0.3200

Note: Robust standard errors are reported in parentheses. ** indicate significance at the 5% levels, respectively.

**Table 5:** Discretized effects of the fertility penalty for women.

Fertility Penalty π	Probability
0%	0.27
18.32%	0.58
74.78%	0.15

**Table 6:** Estimated average child consumption cost.

	$\mu_{parent}$	0 Years Old	1–2 Years Old	3–22 Years Old
Values	0.0577	8216 CNY	8216 CNY	30,391 CNY

**Table 7:** Fitted parameter values for the logarithm of education costs in 2022.

	$\mu_{edu}$	$c_{1}$	$c_{2}$	$c_{3}$	$c_{4}$	$c_{5}$
Values	0.0172	−0.0004	0.0216	−0.3714	2.5019	3.3480

**Table 8:** Summary of parameter fitting.

Symbol	Description	Value
Preference		
γ	Relative risk aversion coefficient of consumption	2.2
κ	Relative risk aversion coefficient of total number of children	0.81
ζ	Parental preference for children	4.2
λ	Negative consumption penalty coefficient	70
β	Utility discount factor	0.99
ξ	Bequest motive	70
x	Initial age of the family	25
$x_{F}$	Maximum childbearing age	49
$x_{R}^{M}$	Husband’s retirement age	60
$x_{R}^{F}$	Wife’s retirement age	55
$x_{T}$	Maximum age	90
Housing expenditure and child-rearing expenditure		
χ	Down payment ratio for home purchase	30%
∆H	Increase in housing space demand with the addition of one child	13.076
$r_{m}$	Mortgage interest rate	3.5%
$\mu_{edu}$	Education cost growth rate	1.72%
$\mu_{parent}$	Child consumption cost growth rate	5.77%
Wages and pension plans		
$\mu_{M}$	Male wage growth rate over time	8.12%
$\mu_{F}$	Female wage growth rate over time	6.34%
$\vartheta^{M}$	Impact of short-term income shocks on male earnings	0.7923
$\vartheta^{F}$	Impact of short-term income shocks on female earnings	0.8047
${\sigma_{\upsilon }^{M}}^{2}$	Variance of male income related to age	0.8012
${\sigma_{\upsilon }^{F}}^{2}$	Variance of female income related to age	0.6161
${\sigma_{\varepsilon }^{M}}^{2}$	Variance of male income related to time period	0.1922
${\sigma_{\varepsilon }^{F}}^{2}$	Variance of female income related to time period	0.3683
$\delta^{M}$	Male pension replacement rate	46%
$\delta^{F}$	Female pension replacement rate	40%
r	Savings interest rate	1.5%
Estimation of the effect of fertility penalty		
$P_{1}$	Probability of no reduction in wages	0.27
$P_{2}$	Probability of a 18.32% reduction in wages	0.58
$P_{3}$	Probability of a 74.78% reduction in wages	0.15

**Table 9:** Numerical comparison of fertility intentions under different levels of fertility penalties.

	Baseline Scenario	Scenario 1	Scenario 2	Scenario 3
Average number of children per family at age 49	1.4196	1.6136	1.5273	1.3423
Proportion of families with two or more children	41.96%	61.36%	53.73%	34.23%
Age at which families stop making fertility decisions	35	38	37	34

**Table 10:** Numerical comparison of fertility intentions under different career interruption durations.

	Baseline Scenario	Scenario 4	Scenario 5	Scenario 6
Average number of children per family at age 49	1.4196	1.4137	1.3703	1
Proportion of families with two or more children	41.96%	41.37%	37.03%	0%
Age at which families stop making fertility decisions	35	35	34	35

**Table 11:** Numerical comparison of fertility intentions under different income and childrearing costs.

	Baseline Scenario	Scenario 7	Scenario 8	Scenario 9
Average number of children per family at age 49	1.4196	1.8213	1.9489	1.3264
Proportion of families with two or more children	41.96%	82.13%	94.89%	32.64%
Age at which families stop making fertility decisions	35	42	40	30

**Table 12:** Numerical comparison of fertility intentions under different education years.

	Baseline Scenario	Scenario 10	Scenario 11	Scenario 12
Average number of children per family at age 49	1.4196	1.3692	1.2698	1.1344
Proportion of families with two or more children	41.96%	36.92%	26.98%	13.44%
Age at which families stop making fertility decisions	35	35	35	34

**Table 13:** Numerical comparison of different subsidy forms.

	Baseline Scenario	Scenario 13	Scenario 14	Scenario 15
Average number of children per family at age 49	1.4196	1.4196	1.4277	1.6275
Proportion of families with two or more children	41.96%	41.96%	42.77%	62.75%
Age at which families stop making fertility decisions	35	35	35	39

**Table 14:** Numerical comparison of effects under different childcare costs.

	Baseline Scenario	Scenario 16	Scenario 17	Scenario 18
Average number of children per family at age 49	1.4196	1.4415	1.4264	1.4650
Proportion of families with two or more children	41.96%	44.15%	42.64%	46.50%
Age at which families stop making fertility decisions	35	35	35	35

**Figure A1:**
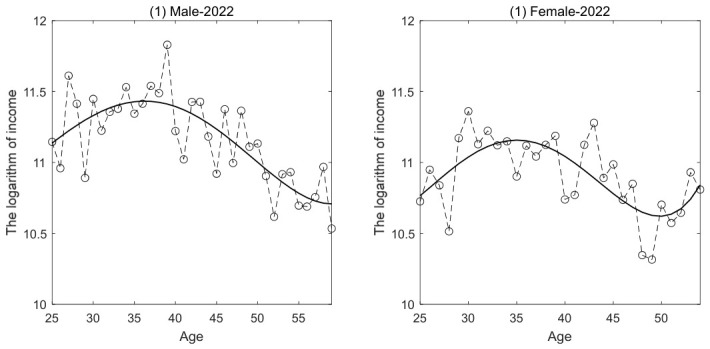
Observed (circles) vs. fitted (curves) log income for males and females.

**Figure A2:**
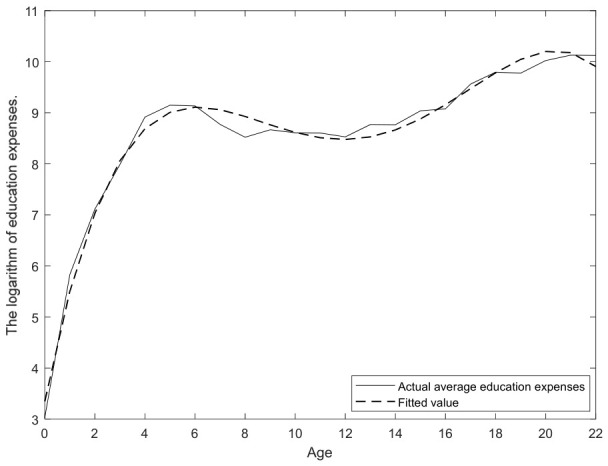
Fitting results of education costs.

**Figure A3:**
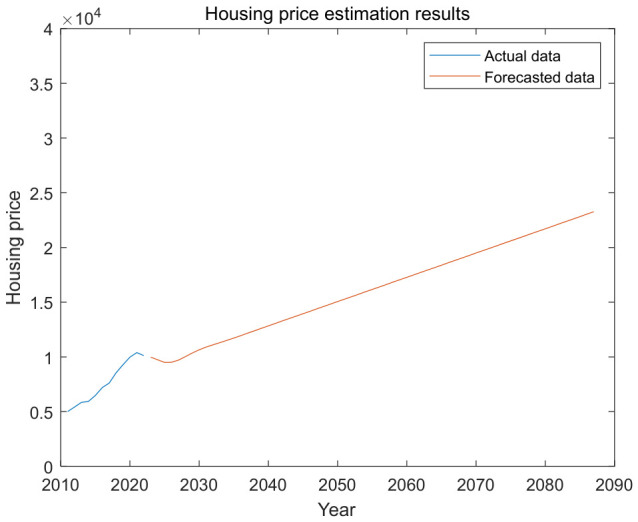
Housing price estimation results.

## Data Availability

The data presented in this study are derived from the China Family Panel Studies (CFPS), a publicly accessible database. The CFPS datasets are available from the Institute of Social Science Survey (ISSS) at Peking University (https://www.isss.pku.edu.cn/cfps/, accessed on 15 March 2024). All data used in this study are anonymized and can be accessed by researchers through the official CFPS data application process.

## Notes

1 DellaVigna (2018) systematically elucidates the research advantages and empirical implementation of structural estimation within behavioral economics. Utilizing structural estimation in their study of individual disappointment-averse preferences, Gill and Prowse (2012) quantify the aggregate degree of disappointment aversion while identifying heterogeneous variations across cohorts. In a lifecycle framework, Laibson et al. (2026) applies structural estimation to estimate β-δ time preferences and coefficients of relative risk aversion, effectively explaining the co-existence of preretirement wealth accumulation and frequent credit card consumption among households.

2 Under an ideal empirical setting, women’s opportunity costs would be measured using continuous indicators such as fertility-induced wage declines or the duration of career interruptions. However, micro-level survey datasets such as the CFPS do not provide direct longitudinal information on women’s income trajectories before and after childbirth. Given these data limitations, this study employs a binary proxy variable indicating whether a woman left the labor market due to childbirth or other family-related reasons (i.e., a reduction of labor income to zero) to capture women’s opportunity costs. Labor market exit represents one of the most direct and substantial forms of career interruption and reflects the significant labor market costs associated with childbirth. Therefore, this proxy retains considerable empirical relevance and practical feasibility for the purposes of this study.

3 Given the strong interpretability of OLS estimates and their convenience for directly comparing changes in the coefficients of the core explanatory variable across different model specifications, this study adopts the OLS estimation results as the benchmark model. In the subsequent robustness checks, alternative econometric models are employed, yielding qualitatively similar results.

4 This study constructs the key explanatory variable based on Question KG23 in the CFPS 2022 self-response questionnaire: “What was the main reason for leaving your most recent job?” When respondents selected “childbirth or other family-related reasons,” the women’s opportunity cost variable was coded as 1; otherwise, it was coded as 0.

5 Following the Ministry of Education’s Kindergarten Work Regulations, this study assumes that children enter kindergarten at age 3 years, after which their need for caregiving declines substantially.

6 Incorporating housing costs into rigid child-rearing costs is primarily based on the consideration that our model focuses on fertility decision making, emphasizing factors influencing the decision itself rather than the actual child-rearing process after childbirth. If people anticipate being unable to provide adequate housing conditions after having children, they tend to avoid childbearing. Therefore, it is reasonable to treat housing expenses as rigid expenditures in the decision-making process.

7 The detailed calculation method is provided in the Appendix A.

8 It is worth noting that variations in the informational environment and idiosyncratic psychological traits can lead to a divergence in these subjective parameters (Solodoha, 2024; Chernyak-Hai & Solodoha, 2026). Although the present study treats the informational environment as an exogenous variable and does not explicitly incorporate individual psychological heterogeneity, endogenizing these two dimensions within the theoretical framework represents a vital and promising direction for our future research.

9 DellaVigna (2018), Gill and Prowse (2012), and Laibson et al. (2026) all utilize structural estimation approaches to calibrate individual behavioral parameters. Their works provide established methodologies for operationalizing “psychological perceptions” from behavioral economics within mathematical models.

10 According to the 2023 China Blue-Collar Employment Research Report, the average monthly wage of maternity nannies is 8824 CNY, which exceeds the average wage level of urban women.

11 If the mother experiences a career interruption while the child is aged 0–2, it is assumed that she provides full-time care at home; thus, no childcare fees are incurred.

12 At first glance, the model specification would imply a 0.15 probability of a 112.17% wage reduction. However, for computational implementation, the program defaults to interpreting this as a 0.15 probability of a 100% wage reduction.

13 This result differs slightly from real-world observations. In reality, some women leave the labor market after the birth of their first child and become full-time homemakers, yet continue to have additional children. This often occurs because their spouses have sufficiently high earnings to cover the childrearing costs of multiple children. In contrast, this study calibrates male income based on the population average, which is insufficient to sustain the expenses associated with raising more than one child. Consequently, under the model specification, women who permanently exit the labor market do not continue to have additional children.

14 According to CFPS 2022 statistics, the annual per capita income of rural residents is 42,663 CNY, which corresponds to 71.38% of the urban per capita wage (59,773). In contrast, residents of China’s four municipalities (Beijing, Tianjin, Shanghai, and Chongqing) have an annual per capita income of 88,756 CNY, equivalent to 148.49% of the urban per capita wage. Based on these figures, this study sets the income and childrearing costs of rural households to 70% of the baseline level (urban households), and those of large-city households to 150% of the baseline level.

15 Data source: https://www.qstheory.cn/dukan/qs/2022-08/01/c_1128878530.htm (accessed on 27 May 2026).

16 Data source: https://www.caf.fr/ (accessed on 15 March 2024).

17 According to the Standard of Fees for Public-run Kindergartens in Beijing, the monthly care and education fee for a “no-level” kindergarten in Beijing shall not exceed 250 CNY; for a first-level kindergarten, the care and education fee shall not exceed 750 CNY; the accommodation fee shall not exceed 300 CNY per month.

## Appendix A

### Appendix A.1. Wage Process and Pension Replacement Rate

The estimation of wages and pension replacement rates was performed through the following steps. First, referring to Cho et al. (2021), the average wage functions for males and females as a function of age are estimated. Then, following Heathcote et al. (2010) and Tonetti (2011), the wage growth rates $\left\{\mu_{M},\mu_{F}\right\}$ for males and females are estimated using the method of simulated moments. Next, based on Heathcote et al. (2010), Tonetti (2011), and Tauchen (1986), the stochastic component of wages is estimated and discretized into five states. Finally, the pension replacement rate is estimated based on current pension policies.

This study uses urban male and female data from the China Family Panel Studies (CFPS) to estimate the average wages of males and females as a function of age. Following Cho et al. (2021), a fourth-degree polynomial is used to fit the deterministic component of wages, and the results are shown in Figure A1 below.

The fourth-degree polynomial fitting results for the wage income $ln{\bar{w}}_{a}^{M}$ and $ln{\bar{w}}_{a}^{F}$ are shown in Table A1.

**Table A1:** Polynomial fitting parameters for average wage income (logarithm) in 2022.

Gender	$c_{1}^{i}$	$c_{2}^{i}$	$c_{3}^{i}$	$c_{4}^{i}$	$c_{5}^{i}$
i=M	0.000003	−0.000410	0.019796	−0.351639	12.871136
i=F	0.000011	−0.001535	0.075408	−1.523009	21.399966

Subsequently, this study utilizes data from CFPS 2018, CFPS 2020, and CFPS 2022, referencing methodologies from Heathcote et al. (2010) and Tonetti (2011), to estimate the average wage growth rates for males and females $\left\{\mu_{M},\mu_{F}\right\}$ via the minimum distance method. Income data from 2018 and 2020 are adjusted to 2022 price levels using China’s 2018–2020 inflation data. We define the actual log-wage change over time as $\nabla_{a,t}=log{\bar{w}}_{a,t+t_{0}}-log{\bar{w}}_{a,t_{0}}$, where the fitted change based on growth rates over period ∆t is $M\left(\mu ,∆t\right)=\mu \times ∆t$, and the observed change is $\hat{M}\left(a,\mu ,∆t\right)=\nabla_{a,t+∆t}-\nabla_{a,t}$. By solving the minimization problem, $\underset{\mu }{\min}\sum_{a,∆t}{\left[\hat{M}\left(a,\mu ,∆t\right)-M\left(\mu ,∆t\right)\right]}^{2}$, we derive the optimal wage growth rate μ. Separate estimations for male and female wage data yield $\mu_{M}=0.08$ and $\mu_{F}=0.06$.

Subsequently, drawing on methodologies from Heathcote et al. (2010), Tonetti (2011), and Tauchen (1986), this study estimates the stochastic component of wages, with estimation results presented in Table A2. Additionally, following Tonetti (2011), we discretize wages into five states.

**Table A2:** Fitted parameter values for wage volatility.

Gender	$\mu_{i}$	$\vartheta^{i}$	${\sigma_{\upsilon }^{i}}^{2}$	${\sigma_{\varepsilon }^{i}}^{2}$
i=M	0.0812	0.7923	0.8012	0.1922
i=F	0.0634	0.8047	0.6161	0.3683

Finally, referencing China’s current basic pension insurance system—specifically, the State Council Decision on Improving the Enterprise Employee Basic Pension Insurance System (State Council Document No. 38 [2005])—this study sets pension-related parameters and calculates replacement rates. Institutional rules stipulate that employers contribute 20% of an individual’s wage income to a pooling account, whereas individuals contribute 8% to a personal account. The pooling account provides a basic pension, calculated as 1% of the accrual base per year of contributions, and the personal account disburses a personal pension equal to the account balance at retirement divided by an actuarial divisor (e.g., 139 months). Based on these rules, the calculated pension replacement rates are 46% for urban male workers ($\delta^{M}=46\%$) and 40% for urban female workers ($\delta^{F}=40\%$).

### Appendix A.2. Child Consumption Costs

Children aged 0–2 have not yet entered preschool and exhibit a high degree of dependence on parental care. Their dietary patterns and daily needs also differ substantially from those of older children. Consistent with existing studies (Letablier et al., 2009; Brandrup & Mance, 2010), this study approximates the consumption cost of newborns by calculating the change in total household consumption expenditure before and after the arrival of an additional infant, thereby estimating the child consumption cost for ages 0–2. The specific procedure is as follows. First, household identifiers in CFPS 2020 and CFPS 2022 are matched. Second, households whose size increased from “two to three members” or “three to four members” are selected. Finally, for these households, the difference in “residential consumption expenditure” across the two waves is computed, and the mean value of these differences is taken as the annual childrearing cost for ages 0–2. Based on this approach, this study sets the annual child consumption cost for ages 0–2 at 8216 CNY.

In the China Fertility Cost Report 2024, it points out that children aged 3–22 exhibit dietary and daily consumption patterns similar to those of adults. Accordingly, the child consumption cost for ages 3–22 can be estimated using the per capita consumption expenditure of adults. According to the China Statistical Yearbook 2023, the per capita annual consumption expenditure in 2022 was 30,391 CNY. Therefore, this study sets the annual child consumption cost for ages 3–22 at 30,391 CNY.

This study estimates post-22-year-old child-rearing costs using data from CFPS 2022, specifically the “Amount of Financial Support Provided by Father” and “Amount of Financial Support Provided by Mother.” Statistical results indicate that the vast majority of adult children receive less than 1000 CNY in parental financial assistance. Therefore, this study sets the parental child-rearing costs for children over age 22 to 0.

Additionally, this study accounts for the annual growth of child consumption cost by estimating their escalation rate. Based on triennial data from CFPS 2018, 2020, and 2022, we employ the minimum distance method to estimate the real growth rate of per capita consumption expenditure (inflation-adjusted) at 5.77%, which is applied as the growth rate for child consumption costs.

Therefore, the parameters related to child-rearing costs are as shown in Table A3.

**Table A3:** Estimated average child consumption cost.

	$\mu_{parent}$	0 Years Old	1–2 Years Old	3–22 Years Old
Values	0.0577	8216 CNY	8216 CNY	30,391 CNY

### Appendix A.3. Education Costs

Similarly to the wage process estimation, this study employs quartic polynomial fitting to model education costs. Using data from CFPS 2022—specifically, the “Total Educational Expenditure over the Past 12 Months (yuan)” field—the quartic polynomial fitting results for education costs are as follows.

As shown in Figure A2, children’s education costs generally increase with age and exhibit a bimodal pattern. This trend reflects the changing patterns of education costs across different stages. From ages 0 to 5, households rapidly increase spending on learning materials and tuition fees, reaching the first peak at age 5. From ages 6 to 14, as children enter upper primary and junior secondary school, education costs remain relatively stable. After age 14, as children face academic pressures from high school entrance exams and high school studies, expenditures resume growth due to purchases of specialized learning resources and training courses. Around age 20, when children enter university, education costs reach the second peak. Post-graduation (after age 22), this study assumes no further education costs.

Finally, accounting for the annual growth of education costs, this study estimates the growth rate of educational expenditures using the minimum distance method, based on triennial survey data from CFPS 2018, 2020, and 2022. The estimation results are presented in Table A4.

**Table A4:** Fitted parameter values for the logarithm of education costs in 2022.

	$\mu_{edu}$	$c_{1}$	$c_{2}$	$c_{3}$	$c_{4}$	$c_{5}$
Values	0.0172	−0.0004	0.0216	−0.3714	2.5019	3.3480

### Appendix A.4. Housing Costs of Childbirth

This study empirically estimates the marginal housing demand associated with childbearing. Specifically, household housing area (housingsize) is set as the dependent variable, with the total number of household members (people) as the core explanatory variable, while controlling for the logarithm of household income (lnincome), mean household age (age), squared mean household age (age2), debt status (debt, a 0–1 dummy variable), and health status (health, a 0–1 dummy variable). The regression results are presented in Table A5.

**Table A5:** Regression results for housing size.

Explanatory Variables	Correlation Coefficient	Standard Deviation
people	13.07616 ***	2.152455
age	−6.23104	4.435288
age2	0.095183	0.059539
lnincome	16.89955 ***	3.973918
debt	7.679501	7.834284
health	−3.54054	9.424805
constant	24.92077	81.57275

Note *** indicate significance levels of 1%, respectively.

The regression results indicate that household housing area is positively correlated with the number of household members at the 1% significance level, with a coefficient of 13.076. Accordingly, this study assumes that each additional family member increases housing demand by 13.076 square meters. Furthermore, referencing China’s mortgage regulations, we set the down payment ratio at χ=30% and the mortgage interest rate at $r_{m}=3.5\%$.

Finally, drawing on methodologies from Hu (2022) and Fu et al. (2021), this study employs an ARIMA model to forecast future housing price trends. First, the data undergoes first-order differencing to achieve stationarity. Following the Akaike information criterion (AIC), the model is specified as ARIMA (3, 1, 3). The projected housing prices for 2023–2057 are illustrated in Figure A3.

Finally, incorporating the above estimation results, suppose the household gives birth to the k-th child in period $t_{k}(k=1,\;2,\;3)$, when the household is aged $x+t_{k}$. The per-period housing cost associated with the k-th child, denoted by $he_{x+t}^{k}$, can then be expressed as follows:

(A1) $${he}_{x+t}^{k}=\left\{\begin{array}{cc} {\chi \cdot ∆H\cdot p}_{x+t_{k}} & t=t_{k}\\ \frac{{\left(1-\chi \right)\cdot ∆H\cdot p}_{x+t_{k}}}{\sum_{k=1}^{x_{R}^{M}-\left(x+t_{k}+1\right)}{\left(1+r_{m}\right)}^{-k}} & t_{k}<t<x_{R}-x\\ 0 & t\geq x_{R}-x \end{array}\right.$$

Here, χ denotes the down payment ratio for home purchases; $p_{x+t}$ represents the housing price in period t; $r_{m}$ indicates the mortgage interest rate; and $x_{R}^{M}$ signifies the retirement age for males.

### Appendix A.5. Structural Estimation of Subjective Parameters

This study calibrates household preference parameters using the simulated method of moments (SMM) and compares model outputs with actual fertility data. Specifically, an initial set of household preference parameters $\Theta =\left\{\gamma ,\beta ,\kappa ,\zeta ,\xi ,\lambda \right\}$ is hypothesized. Monte Carlo simulations are then used to generate 100,000 household life-cycle paths. For each path, household fertility decisions are computed, and the average number of children per household is derived. The discrepancy $g_{x+t}\left(\Theta \right)$ between model results and empirical data is calculated. This process is iteratively repeated until the gap is sufficiently minimized.

The difference between the model’s single-period result and the actual result, $g_{x+t}\left(\Theta \right)$, is defined as follows:

(A2) $$g_{x+t}\left(\Theta \right)=\frac{1}{N}\sum_{i=1}^{N}{\tilde{n}}_{i,x+t}-\frac{1}{K_{x+t}}\sum_{i=1}^{K_{x+t}}{\hat{n}}_{i,x+t}$$

The optimization process can be formulated as follows:

(A3) $$\underset{\Theta }{\min}\sum_{t=0}^{x_{F}-x}{W_{x+t}\left[g_{x+t}\left(\Theta \right)\right]}^{2}$$

where $W_{x+t}=\frac{K_{x+t}}{\sum_{t=0}^{x_{F}-x}K_{x+t}}$ denotes the weighting factor for households aged x+t in the calibration process, reflecting their proportional representation in the cross-sectional data relative to the total household population.

After solving the minimization problem (3), this study obtains the estimated values of the subjective parameters Θ, as shown in Table 11, and summarizes all parameter estimates in Table A6.

**Table A6:** Estimation results of subjective parameters.

	γ	β	κ	ζ	ξ	λ
Values	2.2	0.99	0.81	4.2	70	70
